# Reliability and Validity of the Anterior Knee Pain Scale: Applications for Use as an Epidemiologic Screener

**DOI:** 10.1371/journal.pone.0159204

**Published:** 2016-07-21

**Authors:** Richard F. Ittenbach, Guixia Huang, Kim D. Barber Foss, Timothy E. Hewett, Gregory D. Myer

**Affiliations:** 1 Division of Biostatistics and Epidemiology, Cincinnati Children’s Hospital Medical Center, Cincinnati, Ohio, United States of America; 2 Department of Pediatrics, University of Cincinnati College of Medicine, Cincinnati, Ohio, United States of America; 3 Division of Sports Medicine, Department of Pediatrics, Cincinnati Children’s Hospital Medical Center, Cincinnati, Ohio, United States of America; 4 Mayo Clinic Biomechanics Laboratories and Mayo Sports Medicine Center, Rochester and Minneapolis, Minnesota, United States of America; 5 Department of Orthopaedic Surgery, University of Cincinnati, Cincinnati, Ohio, United States of America; 6 Sports Health & Performance Institute, The Ohio State University, Columbus, Ohio, United States of America; 7 The Micheli Center for Sports Injury Prevention, Waltham, Massachusetts, United States of America; Queen's University, CANADA

## Abstract

A screening instrument’s ability to provide clinicians with consistent and reproducible information is crucial to intervention. Despite widespread acceptance and clinical use of the Kujala Anterior Knee Pain Scale (AKPS) in orthopedics and sports medicine, few studies have reported on its reliability and no such studies have concentrated on child or adolescent samples exclusively, segments of the population for which this instrument is often used. The purpose of the current study was to describe and report on the reliability and validity of the AKPS for use with high school female athletes participating in interscholastic athletics. The study was a secondary analysis of prospective epidemiologic data using established scale validation methods. The records of 414 female athletes 11.0 to 18.1 years of age (Mean 13.9 yrs, SD = 1.7 yrs) were used for analysis. Four different approaches to scoring and scale reduction of the AKPS were evaluated, including the original, ordinal 13-item form, a modified, ordinal 6-item form, a modified, dichotomous 13-item form, and a modified, dichotomous 6-item form. Three different types of reliability (internal consistency, equivalence across forms, standard error of measurement) and one type of validity (criterion-related) were estimated for the AKPS in the current sample. The four scoring formats of the AKPS scale were found to have high internal consistency (α_coef_ = 0.83 to 0.91), equivalence across the short and long forms (*r* = 0.98), acceptable standard errors of measurement (0.82 to 3.00), and moderate to high criterion related validity—as determined by physican’s diagnosis: 0.92 (13-item form), 0.90 (6-item form). The Kujala AKPS is a valid and reliable measure of anterior knee pain and appropriate for use as an epidemiologic screening tool with adolescent female athletes.

## Introduction

Within the fields of orthopedics and sports medicine, the Kujala Anterior Knee Pain Scale (AKPS) [1], has been widely used to identify and study the prevalence of patellofemoral knee pain. However, despite its widespread acceptance clinically, relatively few studies have reported on its technical properties with pediatric patients. Only two studies have focused on the measurement properties of patellofemoral pain instruments, in general [2, 3], and only one study has focused on the technical properties of the AKPS, specifically [4]. Whereas Bennel et al. [4] and Crossley et al. [2] have reported reliability information for the AKPS, both were conducted using small, narrowly defined samples of adults. No studies to date appear to have taken a comprehensive view of reliability assessment and none has been conducted using pediatric or adolescent samples. Even the two studies that used translations into other languages were done using adult groups and with specific disease states [5, 6]. This lack of evidence regarding the AKPS in the professional literature, and its potential to provide clinicians with specific reliability information on the symptomatic evaluation of anterior knee pain in large samples of adolescents leaves an important gap in the pediatric sports medicine literature. Consequently, the purpose of this study was to describe and report on the reliability and validity of the AKPS with adolescent female athletes participating in interscholastic athletics.

## Materials and Methods

### Sample

The records of 414 adolescent girls 11.0 to 18.1 years of age (Mean 13.9 yrs, SD = 1.7 yrs) were analyzed as a secondary analysis of a prospective epidemiologic study. The study was designed to assess patellofemoral pain in a diverse, non-clinical sample of adolescent female athletes who participated in three interscholastic sports programs, namely basketball, soccer, and volleyball, within a single school district in rural Kentucky (3.9% African American, 94.0% European American (White), 1.0% Asian and 1.2% Hispanic). See Myer et al. [7] for a more complete description of the sample and methods used to obtain the primary data. Signed written informed consent was obtained from the parent/guardian of all participants under the age of 18. Participants 18 years of age and older signed an adult written consent form. The study was approved by Cincinnati Children’s Hospital Institutional Review Board.

### Instrument

The Kujala AKPS [1] is a 13-item screening instrument designed to assess patellofemoral pain in adolescents and young adults, with a variable ordinal response format. For example, a ‘Limp’ score would be scored as follows: none (5), slight/periodic (3), constant (0). Total scores range from 0 to 100. Myer et al. have offered a 6-item short form based on simplified, dichotomously recoded items. As such, a recoded ‘Limp’ score would be recoded as: none (0), slight/periodic/constant (1).

### Statistical Analyses

Three different sets of reliability estimates were computed for the AKPS for the entire sample of 414 research participants: internal consistency (inter-item similarity), equivalence (comparability across short and long forms), and standard error of measurement (potential variability in true scores), as well as an estimate of criterion-related validity, comparison of AKPS scores with physicians’ diagnosis of patellofemoral knee pain.

First, Cronbach’s alpha (α_Coef_) was used to estimate internal consistency among items. Second, Spearman-rho correlation coefficients were used to estimate the equivalence of scales between the 6-item brief forms with their longer, 13-item forms. Third, standard error of measurement (SEM) values were calculated for each set of scores, with SEM defined as the likelihood of a score to vary about its true mean, and calculated as follows: s^2^_E_ = s_x•_(1 –r_xx_)^0.5^ where s^2^_x_ is defined as the error variance of the knee pain scores, s_x_ = standard deviation of the scores, and xx = internal consistency of the AKPS test (also defined as α_Coef_). With respect to validity, and criterion-related validity in particular, the AKPS’s ability to match physician diagnosis was evaluated for comparability across both short and long forms, under pre- and post-test conditions, using four distinct samples of high-school athletes (i.e., exclusively healthy, healthy who become injured, injured who become healthy, and exclusively injured).

Traditional test-retest reliability analyses were not deemed appropriate given the changing nature of the samples with respect to injuries, the length and complexity of seasons with respect to additional sports and activities, as well as the restricted nature of the scores for segments of the sample (e.g., ceiling effect of healthy scores). Throughout all analyses described here, the original 13-item (long) form of the AKPS was evaluated as well as the 6-item short form identified by Myer et al. [7] in both the ordinal and dichotomized scoring formats. All analyses were descriptive in nature, used a pre-specified statistical analysis plan approved by the entire team, and computed using SAS v9.3 software.

## Results

### Internal Consistency

Measures of internal consistency were computed for both the 13-item long form as well as the 6-item short form for both ordinal and dichotomously recoded response option formats: ordinal 13-item form (α_Coef_ = 0.91), dichotomous 13-item form (α_Coef_ = 0.91); ordinal 6-item form (α_Coef_ = 0.84), dichotomous 6-item form (α_Coef_ = 0.83; Table 1).

**Table 1:** Scale Level Summary Statistics (*N* = 414).

Response Options	*M* (*SD*)	Range	Internal Consistency	Alternate Forms^†^	SEM^‡^
**Ordinal**					
13-item	94.56 (9.68)	33, 100	0.91	0.98	3.00
6-item	47.28 (5.01)	18, 50	0.84		1.20
**Dichotomous**					
13-item	2.08 (3.32)	0, 13	0.91	0.98	1.50
6-item	1.05 (1.66)	0, 6	0.83		0.82

^†^Alternate forms reliability estimated for both the 13-item and 6-item forms.

^‡^Standard deviation estimates used to compute the aforementioned SEMs were as follows: Ordinal, 13-item form (*SD* = 10); Dichotomous, 13-item form (*SD* = 5); Ordinal, 6-item form (*SD* = 3); Dichotomous, 6-item form (*SD* = 2), and were drawn from both the literature as well as our current sample.

### Equivalence

Alternate forms reliability of the ordinal 13-item form with the ordinal 6-item form (*r* = 0.98), and the dichotomous 13-item form with the dichotomous 6-item form (*r* = 0.98), were identical.

### Standard error of measurement (SEM)

Variability associated with the errors of test scores was computed as follows: ordinal 13-item form SEM = 3.00, dichotomous 13-item form SEM = 1.50, ordinal 6-item form SEM = 1.20, dichotomous 6-item form SEM = 0.82.

### Validity

Criterion-related validity was evaluated by comparison of the AKPS’s ability to map *consistently* onto physician’s diagnosis of athletes’ knee pain for pre-test and post-test scores, using simple % correct classification. In general, ‘correct’ classification rates for the 6-item format were higher than for the 13-item format (see Table 2). The dichotomously scored items manifested identical classification rates to those of the ordinal response format. For exclusively healthy or exclusively injured groups, the median classification rates remained very high, at 86% (healthy) and 99% (injured), respectively, and never varied by more than 2% across observation periods.

**Table 2:** Percent Correct Identification of AKPS Score when compared with Physical Diagnosis (*N* = 414).

Scoring Format^†^	13-Item Form		6-Item Form	
	Pre-test	Post-test	Pre-test	Post-test
Healthy-Healthy	0.84	0.85	0.86	0.88
Healthy-Injured	0.68^‡^	0.98	0.72^‡^	0.92
Injured-Healthy	1.00	0.64^‡^	0.97	0.67^‡^
Injured-Injured	1.00	0.98	1.00	0.98

^†^Subgroup sample sizes are as follows: Healthy-Healthy *n* = 273; Healthy-Injured *n* = 40; Injured-Healthy *n* = 36; Injured-Injured *n* = 65.

^‡^Denotes lower classification rate for ‘healthy’ or ‘injured’ athletes when changing status. Misclassification rates are defined as 1 - % correct classification.

Among athletes who changed state over the course of the season (healthy to injured or injured to healthy), the AKPS appeared to over-estimate the number of healthy athletes with knee pain (false positive) more often than it injured athletes as healthy (false-negative). For example, when used with adolescents who went from healthy to injured status, the median classification rates went from 72% to 92%, and when used with athletes who went from injured to healthy, the median classification rates went from 97% to 67%. The lone exception to higher correct classification rates for the 6-item as compared to the 13-item format was for athletes who became injured over the course of the season, when the AKPS’s classification rate fell from 98% (13-item format) to 93% (6-item format).

## Discussion

The Kujala AKPS, is a well-recognized and highly respected instrument used within the fields of orthopedics and sports medicine. Less well developed, however, is the psychometric foundation on which the instrument is based. The purpose of this study was to describe and report on the reliability and validity of the AKPS using both the original 13-item form and a more concise 6-item form among a sample of high-school female athletes. Both ordinal and dichotomous response option formats were evaluated using data obtained from a sample of *N* = 414 adolescent females participating in interscholastic athletics. Reliability was estimated using internal consistency, equivalence across forms, and standard error of measurement; validity was evaluated using percent correct classification rates at both pre- and post-season evaluations.

All four scoring formats of the AKPS evidenced high internal consistency (α_Coef_ = 0.83 to 0.91), with the longer 13-item forms having identical internal consistency irrespective of response format (α_Coef_ = 0.91). With respect to equivalence (or alternate forms reliability), the correlation between the long and short forms remained very high (*r* = 0.98), also irrespective of whether one used the dichotomous or the ordinal response option formats. With respect to consistency of AKPS’s scores over the athletic season, the scores were markedly consistent with one another once physician diagnosis was taken into account, as scores differed by only a few percentage points. However, the instrument did have a tendency to overestimate the number of athletes who were injured more so than it did the number of athletes where were healthy, which is indeed preferable in a clinical, epidemiologic study as this one. With respect to the AKPS’s ability to identify an emerging injury in an asymptomatic athlete, a potential false positive (over-estimating) diagnosis of an injured state is preferred over a false negative.

Whereas the 6-item form had identical classification rates to the 13-item form, one may be able to screen at similar accuracy and reliability levels with the shorter form, thereby saving valuable time for clinical care and intervention. In the case of a prospective epidemiologic study, when spread over thousands of athletes, this could mean a tremendous savings to the athletes, the clinicians, and medical and educational systems more broadly. For example, compare the time it takes 2 clinicians to give a 13-item ordinal instrument (~10 min/form) to 1,000 adolescents (total administration time = 333 hours) who may have questions and clarifications needed to complete the form. Compare this to the time it takes the same 2 clinicians to give a 6-item dichotomous (~2 min/form) instrument to the same 1,000 students (total administration time = 67 hours)—time that increases further when the adolescents’, parents’, and/or teachers’ times are taken into account. Shorter instruments with identical psychometric properties can offer clinicians and researchers tremendous advantages, which may include significantly increased study recruitment and decreased study dropout.

The current study is not without limitations. The AKPS was initially designed for use in clinical settings with symptomatic knee pain patients. The current project was part of a prospective, epidemiologic investigation that included a population of healthy, asymptomatic, and minimally symptomatic study participants. While testing this AKPS instrument in expanded populations can enhance its generalizability, the results of populations not included in the instruments’ development should be interpreted with caution. The current cohort captures the entire population of interscholastic female athletes participating in basketball, soccer, and volleyball, in a single Kentucky county; hence, it should be representative of the county’s interscholastic female athlete population participating in those sports. However, the extent to which these findings generalize to athletes of other sports, in other grade levels, or in other U.S. counties, remains undetermined. The instrument’s revised scoring system gave all healthy patients a maximal score, instead of allowing for a range of functional but minimally symptomatic states, which impacted the types of analyses that can be conducted. The analyses performed here were performed with modifications to the scoring system after the instrument had been administered. It will be important for additional research to be conducted with this instrument, to administer the scale in its various forms to the athletes prior to additional use, and to athletes representing broader segments of the U.S. population to fully gauge it potential generalizability across other groups, to male athletes, and across entire the spectrum of socioeconomic levels.

## Conclusion

Current AKPS data using the reduced 6-item form appears to offer highly similar reliability indices to the original but longer 13-item form when either the ordinal or the dichotomized response option formats are considered. More research is needed to confirm these findings across ages, sexes and various activity levels. It is also recommended that new studies be conducted in which the different formats are presented to the athletes and scored using the modified forms presented here, to know with increased certainty how the different scales perform in the reduced or simplified states.

## Supporting Information

S1 File AKPS Data File. (XLS)
